# The Interaction between Diabetes, Body Mass Index, Hepatic Steatosis, and Risk of Liver Resection: Insulin Dependent Diabetes Is the Greatest Risk for Major Complications

**DOI:** 10.1155/2014/586159

**Published:** 2014-08-14

**Authors:** M. G. Wiggans, J. T. Lordan, G. Shahtahmassebi, S. Aroori, M. J. Bowles, D. A. Stell

**Affiliations:** ^1^Hepatopancreatobiliary Surgery, Plymouth Hospitals NHS Trust, Derriford Hospital, Derriford Road, Plymouth, Devon PL6 8DH, UK; ^2^Peninsula College of Medicine and Dentistry, University of Exeter and Plymouth University, John Bull Building, Plymouth, Devon PL6 8BU, UK; ^3^School of Science and Technology, Nottingham Trent University, Nottingham NG1 4BU, UK

## Abstract

*Background*. This study aimed to assess the relationship between diabetes, obesity, and hepatic steatosis in patients undergoing liver resection and to determine if these factors are independent predictors of major complications. *Materials and Methods*. Analysis of a prospectively maintained database of patients undergoing liver resection between 2005 and 2012 was undertaken. Background liver was assessed for steatosis and classified as <33% and ≥33%. Major complications were defined as Grade III–V complications using the Dindo-Clavien classification. *Results*. 504 patients underwent liver resection, of whom 56 had diabetes and 61 had steatosis ≥33%. Median BMI was 26 kg/m^2^ (16–54 kg/m^2^). 94 patients developed a major complication (18.7%). BMI ≥ 25 kg/m^2^ (*P* = 0.001) and diabetes (*P* = 0.018) were associated with steatosis ≥33%. Only insulin dependent diabetes was a risk factor for major complications (*P* = 0.028). Age, male gender, hypoalbuminaemia, synchronous bowel procedures, extent of resection, and blood transfusion were also independent risk factors. *Conclusions*. Liver surgery in the presence of steatosis, elevated BMI, and non-insulin dependent diabetes is not associated with major complications. Although diabetes requiring insulin therapy was a significant risk factor, the major risk factors relate to technical aspects of surgery, particularly synchronous bowel procedures.

## 1. Introduction

Liver failure occurs in up to 32% of patients following liver resection [1–5] and is a major contributor to both morbidity [6] and mortality [7]. Liver resection is technically more difficult in patients with parenchymal liver disease [8] and the risks of liver resection are increased due to impaired hepatic regeneration [9].

Nonalcoholic fatty liver disease (NAFLD) is the commonest cause of liver disease in the West [10] and is also the commonest cause of a sustained rise in serum transaminases in patients with no history of chronic liver disease [11]. NAFLD encompasses steatosis (excess accumulation of triglycerides), steatohepatitis (hepatocyte damage, inflammatory infiltrate, and fibrosis), and cirrhosis [12] and can be demonstrated with routine histological staining. NAFLD is associated with diabetes mellitus and obesity [13, 14] which are also undergoing a global epidemic [15, 16]. However, not all patients with obesity and diabetes develop NAFLD and similarly not all patients with NAFLD suffer either diabetes or obesity [17].

Liver-directed chemotherapy is also associated with hepatotoxicity. Steatohepatitis has been shown to occur in 20% of patients who receive irinotecan and 5% of those who receive fluorouracil (5FU) [18], with a resulting increase in complications after surgery. Oxaliplatin is associated with sinusoidal obstruction syndrome [18, 19]. Recreational alcohol use is also a major cause of hepatic steatosis [20].

A meta-analysis has shown that hepatic steatosis is associated with increased risk of postoperative complications and that moderate and severe steatosis are associated with increased mortality compared to patients with normal liver parenchyma or mild steatosis [21]. However, this analysis is based on four studies, only two of which included both BMI and diabetes in multivariate analyses [8, 22–24]. Obesity, diabetes, and hepatic steatosis often coexist in the metabolic syndrome [25], and the increased risk of operating in the presence of steatosis may be due to associated comorbidity. Diabetes mellitus and obesity are independent risk factors for postoperative complications following other types of major surgery, including infectious [26–28], cardiovascular [28, 29], and renal complications [26, 28, 29]. Furthermore in the four studies included in the meta-analysis heterogeneous definitions of postoperative complications were used, and often relatively minor complications were included. Recently complications after liver surgery have been classified by the Dindo-Clavien system [30], which stratifies severity of complications and allows comparison of outcomes between centres.

The aim of this study was to assess the relationship between the incidence of diabetes, obesity, and hepatic steatosis in patients undergoing liver resection after a period of abstention from alcohol consumption and to determine if these factors are independent predictors of major complications following liver resection, using the Dindo-Clavien system.

## 2. Materials and Methods

A retrospective analysis of a prospectively maintained database of all patients undergoing liver resection between July 2005 and September 2012 was undertaken. Patient characteristics, laboratory data, and intraoperative details were retrieved. BMI was recorded preoperatively and the cohort was divided into three categories: 18.5–24.99 kg/m^2^ (normal), 25–29.99 kg/m^2^ (overweight), and ≥30 kg/m^2^ (obese). Diabetes was categorised according to the requirement for insulin. The presence of preexisting chronic liver disease was confirmed by histology. American Association of Anesthesiologists (ASA) grade was determined by the responsible anaesthetist and the physiologic score calculated according to the POSSUM system [31]. Selected patients were treated with neoadjuvant chemotherapy. All patients underwent preoperative counselling by a nurse specialist where abstention from alcohol was mandated. This instruction was also contained in a patient information sheet. The normal interval from preoperative counselling to surgery in this series is approximately 30 days.

Liver resections were defined according to the Brisbane classification [32] and undertaken using standard techniques, using hepatic inflow occlusion selectively. Major resections were defined as resections of three or more segments. Synchronous liver and bile-duct resections were performed in the presence of hilar cholangiocarcinoma. Radiofrequency ablation was used where small lesions were not accessible for surgical resection.

Major complications were defined as Grade III–V complications using the Dindo-Clavien classification where Grade III complications are those requiring surgical, endoscopic, or radiological intervention, Grade IV includes life threatening complications including organ failure, and Grade V is death [30]. Posthepatectomy liver failure (PHLF) was defined in accordance with the International Study Group of Liver Surgery (ISGLS) [33] as an increased prothrombin time (PT) and serum bilirubin concentration on or after postoperative day five. In patients with preoperatively increased PT or serum bilirubin concentration PHLF was defined as an increasing serum bilirubin concentration and PT on or after postoperative day 5, compared with the values of the previous day. Renal dysfunction was defined as an increase in serum creatinine of ≥1.5-fold from the preoperative baseline, according to RIFLE criteria [34].

Serum biochemistry tests and coagulation assays were performed preoperatively, in the first 24 postoperative hours, and then repeated according to clinical course. The peak measurement of bilirubin, prothrombin time (PT), and creatinine was recorded. Clotting factors were not administered between postoperative days (POD) 1–5. At histological examination the background liver parenchyma at least 1 cm from the tumour edge was assessed for degree of steatosis using the Brunt classification (the proportion of hepatocytes containing fat droplets; 1: <33%, 2: 33–66%, and 3: >66%) [35]. For analysis the data was divided into <33% (mild or none) and ≥33% (moderate or severe).

The minimum postoperative followup was 90 days and mortality was recorded along with details of postoperative intervention and complications.

To determine potential associations between patient characteristics and steatosis and between patient, operative, and histological characteristics and major complications univariate logistic regression or chi-square test at the level of *P* < 0.25 [36] was performed, as appropriate. Significant variables in the univariate analysis were included in the multivariate logistic regression model and were considered to be significant if *P* < 0.05. All analyses were carried out using the statistical package R 2.1.14 [37].

## 3. Results

Of 504 patients treated in the study period, surgery was undertaken for metastatic disease in 358 (71.0%), of whom 308 (61.1%) had colorectal liver metastases. Resections were performed for primary hepatic malignancy in 106 patients (21.0%) including hepatocellular carcinoma in 39 (7.7%) and cholangiocarcinoma in 31 (6.2%) patients. In 40 patients (7.9%) resection was performed for benign tumours. Major resection was undertaken in 299 patients (59.3%). In twenty-three patients a synchronous bowel procedure was performed including 10 colonic resections, 11 small bowel procedures, one gastric resection, and one Whipple's procedure. Fifty-six patients were diabetic (11.1%), of whom 15 were insulin dependent (26.8%). The median BMI of patients undergoing resection was 26 kg/m^2^ (range 16–54 kg/m^2^). Elevated BMI (≥25 kg/m^2^) was noted in 332 patients (65.9%) and 123 patients (24.4%) were obese (≥30 kg/m^2^). Five patients had no BMI recorded and were excluded from analysis. Preoperative liver-directed chemotherapy was used in 168 patients (33.3%). The most commonly used regime was oxaliplatin and capecitabine which was used in 118 patients (70.2%). Irinotecan was used in six patients (3.6%).

**Figure 1:**
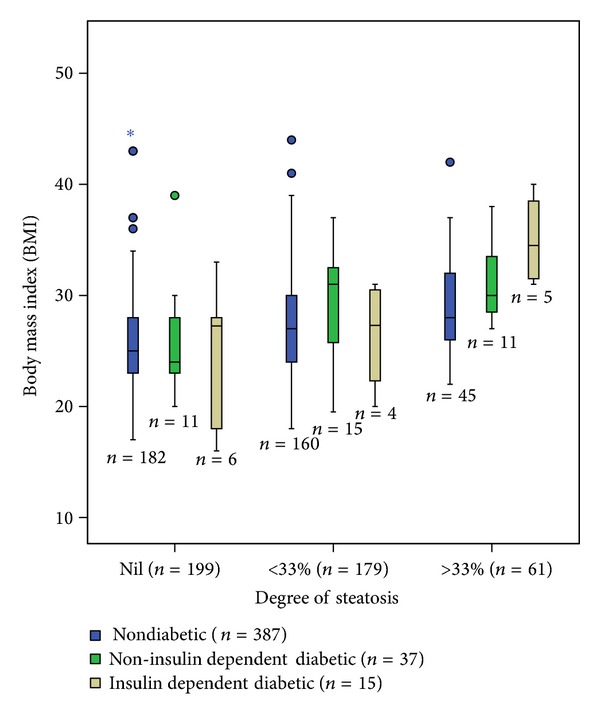
Box plot of body mass index (BMI), diabetic status, and degree of hepatic steatosis in 439 patients undergoing liver resection. Nil versus <33% (*P* < 0.001), <33 versus ≥33% (*P* = 0.001).

Histopathological examination revealed zero, mild, moderate, and severe steatosis in 199 (39.5%), 179 (35.5%), 54 (10.7%), and seven (1.4%) patients, respectively. Degree of steatosis was not recorded in 65 patients (12.9%). The distribution of BMI, diabetes, and steatosis is shown in Figure 1. The median BMI of patients with no steatosis (25 kg/m^2^, range 16–45) was lower than those with mild steatosis (27 kg/m^2^, range 18–44) (*P* < 0.001), which was lower than patients with moderate/severe steatosis (29 kg/m^2^, range 22–42) (*P* = 0.001). The median BMI of diabetic patients was 29 kg/m^2^ (16–40) compared to 26 kg/m^2^ (17–54) in nondiabetic patients (*P* = 0.002). There was no difference in the median BMI of patients with insulin dependent diabetes (IDDM) (29 kg/m^2^, range 16–40) and those with non-insulin dependent diabetes (NIDDM) (29 kg/m^2^, range 20–39) (*P* = 0.816). The rate of mild steatosis among diabetics was 16/52 (30.8%) compared to 45/387 (11.6%) in nondiabetics (*P* = 0.001), but there was no significant difference in the rates of mild steatosis in patients with NIDDM (11/37) and those with IDDM (5/15). The rate of moderate/severe steatosis was 6/135 (4.4%) in normal weight, nondiabetic patients, 39/249 (15.6%) in overweight nondiabetics (*P* = 0.001), 0/12 in normal weight diabetics, and 15/39 (38.5%) in overweight diabetics (*P* < 0.001).

Elevated preoperative transaminase levels were noted in 18 of 60 patients (30%) with moderate/severe steatosis and 61 of 369 patients (16.5%) with steatosis <33% (*P* = 0.019). The sensitivity and specificity of elevated transaminases for predicting the presence of moderate or severe steatosis were 30% and 83%, respectively.

Multivariate analysis revealed that elevated BMI ≥ 25 kg/m^2^ (*P* = 0.001) and the presence of diabetes (*P* = 0.018) were significantly associated with moderate/severe hepatic steatosis (Table 1). BMI ≥ 25 kg/m^2^ increased the risk by a factor of 2.97 and diabetes increased the risk by a factor of 2.69. Among diabetic patients insulin dependence increased the risk of moderate/severe steatosis by a factor of 4.31 (*P* = 0.037). However, BMI ≥ 30 kg/m did not increase the risk of moderate/severe steatosis compared to BMI of 25–29.9 (*P* = 0.144). Raised preoperative transaminase levels also increased the risk of moderate/severe steatosis by a factor of 3.82 (*P* < 0.001), and raised preoperative alkaline phosphatase concentrations decreased the risk by a factor of 0.15 (*P* = 0.001). Hepatic steatosis was not associated with liver-directed chemotherapy or other biochemical markers of liver dysfunction (preoperative hypoalbuminemia and hyperbilirubinemia).

During the study period 94 patients developed a major postoperative complication. Twenty-three patients died within 90 days of surgery (4.6%) and 71 patients who survived beyond 90 days suffered a major complication (14.1%). The most common cause of mortality was liver failure (nine patients).

Of patients who developed Grade IV complications 34/64 (53.1%) developed PHLF and 31/64 developed renal failure (48.4%). Of the 34 patients who developed PHLF 29 had undergone major liver resection. Twenty-three patients developed bile leaks, and seven required relaparotomy/relaparoscopy. Multivariate analysis revealed that older age, male gender, hypoalbuminaemia, synchronous bowel procedures, number of segments resected, and blood transfusion were independent risk factors for major postoperative complications (Table 2). There was no association between NIDDM, BMI, or degree of hepatic steatosis and major postoperative complications. IDDM more than trebled the risk of major complication compared to nondiabetics and those with NIDDM. The complications in these groups are shown in Table 3. The greatest risk however occurred when liver resection was undertaken in conjunction with a synchronous bowel procedure, which increased the risk of major complication almost six times that of a liver-only resection. Ten of 23 patients developed major postoperative complications, six of whom had colonic resections (three right sided and three left sided), three had small bowel procedures, and one had a gastric resection.

In the 299 patients who underwent major resection, there was no significant difference in the proportion of patients with steatosis ≥33% between patients who did (10/64, 15.6%) or did not (23/201, 11.4%) develop major complications (*P* = 0.388). Similarly there was no significant difference in the proportion of patients with steatosis ≥33% between patients who did (4/22, 15.6%) or did not (29/243, 11.9%) develop PHLF (*P* = 0.495).

## 4. Discussion

The principal finding of this study is that although diabetes mellitus and higher BMI are risk factors for steatosis in patients undergoing liver resection, the majority of cases of steatosis occur in nondiabetic patients with mildly elevated BMI (25–30). Secondly, steatosis and elevated BMI are not associated with major complications after liver resection, and diabetes is a risk factor for these complications only if patients are insulin dependent. Other predictors of major complications are older age, male gender, preoperative hypoalbuminaemia, synchronous bowel procedures, number of segments resected, and requirement for blood transfusion.

The 90-day mortality (4.6%) and morbidity (14.7%) rate are similar to published series [4, 18, 38], although other series have included minor (Grade I and II) complications [39–41]. Composite outcomes similar to the one used in this study have been used previously in studies evaluating outcomes following gastrointestinal surgery [42, 43]. The present study confirms the association between hepatic steatosis and BMI [44]. Whilst the rate of moderate/severe steatosis was greatest in overweight diabetic patients (38.5%), it also occurred in patients of normal weight without diabetes (4.4%). This suggests that other risk factors may be involved in the aetiology of the disease. Undernutrition [17], impaired glucose tolerance [45], and genetic factors [46] have also been implicated in the development of NAFLD. Alcohol consumption is an unlikely cause of steatosis in this series as all patients are asked to abstain from alcohol consumption prior to surgery, although compliance with this instruction has not been assessed.

Elevated transaminase levels are associated with hepatic steatosis, but the sensitivity of abnormal transaminases in detecting moderate or severe NAFLD is poor, as 70% of these patients had normal transaminase levels. This is in keeping with other studies [47]. Interestingly, raised preoperative alkaline phosphatase concentration was associated with decreased incidence of steatosis. Elevated alkaline phosphatase may be found in cases of biliary obstruction, and of the 119 patients with this finding 16.8% had cholangiocarcinomas compared to only 2.9% of the 380 patients with normal alkaline phosphatase. This group is more likely to be systemically unwell as a consequence of biliary obstruction and to have suffered a period of anorexia and weight loss, which may affect the degree of hepatic steatosis.

Preoperative chemotherapy was not shown to be associated with steatosis. Studies have shown an association between steatohepatitis and irinotecan therapy [18], which was rarely used in this series. In addition the policy in this unit is to use only four cycles of chemotherapy and to allow a period of recovery before undertaking liver resection, to allow resolution of hepatotoxicity.

Previous studies have shown that steatosis increases the risk of PHLF [8, 21]. The rate of PHLF in this series was low (6.7%) and occurred in 6.6% patients with moderate/severe steatosis and 6.1% of the patients with none/mild steatosis. The majority of cases of PHLF followed major liver resection (29/34). It is possible that there is an independent association between steatosis and PHLF, which is not revealed in this study which uses a composite outcome including other complications in the multivariate analysis. Steatosis may be a risk factor for liver failure in patients undergoing extended hepatectomy, although not in major hepatectomy in this series, where the risk of this complication is greatest. Previous studies have recommended liver biopsy to investigate the presence of steatosis prior to resection [48, 49]. The current study suggests that the risk of this investigation is not justified due to the lack of effect of steatosis on outcome.

The rate of bile leak requiring intervention (4.6%) was not affected by the degree of hepatic steatosis suggesting that hepatic steatosis does not make parenchymal division more difficult to perform.

Elevated BMI was not associated with major complications in this series, although it may be associated with more minor complications such as wound infection which has not been explored in this study.

Diabetes was an independent risk factor for complications after liver surgery which confirms the findings of previous studies [5, 50–52], although identification of insulin dependence as the major risk factor is a novel finding. Whilst there was no significant difference in the risk of major complications between nondiabetic patients and those with non-insulin dependent diabetes, the risk of complications was more than trebled in those with insulin dependent diabetes. This finding reflects the multisystem nature of diabetic end-organ damage. Diabetic nephropathy is a major cause of renal dysfunction [53] and was the most common complication in patients with IDDM. Renal dysfunction was also twice as common amongst patients with IDDM compared to those with NIDDM.

Older age, male gender, preoperative hypoalbuminaemia, number of liver segments resected, and requirement for blood transfusion have all been previously identified as risk factors for postoperative complications [38]. The finding that performing synchronous bowel procedures is associated with worse outcome is similar to that of a previous study which found that the risk of a major complication was 20.4% after a synchronous colonic resection compared to 14.9% after a liver-only resection [54]. Although a recent systematic review suggested no difference in terms of overall morbidity or mortality between synchronous and staged resections [55] the results of the present study reveal the risk of developing a major complication after a synchronous bowel procedure was almost six times that of a liver-only resection. It should also be noted that the synchronous procedures included a gastric resection and Whipple's procedure which may pose different risks to colonic resections. Most of the increased risk in this context relates to leaks from enteric anastomoses.

## 5. Conclusions

The results of this study allow clinicians to advise patients regarding the risks of liver resection and to place them in context. In particular, liver surgery in the presence of steatosis, elevated BMI, and NIDDM does not lead to greatly increased operative risk. While insulin dependence is a significant risk factor for complications after liver surgery, the major risk factors in this series related to technical details of the operation, particularly the performance of simultaneous bowel procedures.

## Figures and Tables

**Table 1:** Analysis of factors associated with hepatic steatosis (≥33%) in 439 patients undergoing liver resection.

*N* = 439	Steatosis < 33% (*n* = 378)		Steatosis ≥ 33% (*n* = 61)		Univariate	Multivariate		
	Median (range)	Count (%)	Median (range)	Count (%)	*P* value	Comparison	Odds ratio (95% CI)	*P* value
Age	65 (21–90)		65 (41–87)		0.622			
Gender								
Female		168 (44.4)		24 (39.3)	0.544			
Male		210 (55.6)		37 (60.7)				
Liver-directed chemotherapy								
Yes		132 (34.9)		20 (32.8)	1.000			
No		246 (65.1)		41 (67.2)				
Preexisting chronic liver disease								
Yes		6 (1.6)		3 (4.9)	0.228∗			0.869
No		372 (98.4)		58 (95.1)				
Preoperative jaundice (≥50 micromoles/L)								
Yes		5 (1.3)		0	0.800			
No		373 (98.7)		61 (100)				
Hypoalbuminaemia (<35 g/L)								
Yes		15 (4.0)		1 (1.6)	0.602			
No		360 (95.2)		59 (96.7)				
Not recorded		3 (0.8)		1 (1.6)				
Raised preoperative alkaline phosphatase								
Yes		92 (24.3)		5 (8.2)	0.008∗		0.15 (0.05–0.46)	0.001∗∗
No		283 (74.9)		55 (90.2)				
Not recorded		3 (0.8)		1 (1.6)				
Raised preoperative transaminase								
Yes		61 (16.1)		18 (29.5)	0.021∗		3.82 (1.85–7.89)	<0.001∗∗
No		308 (81.5)		42 (68.9)				
Not recorded		9 (2.4)		1 (1.6)				
Diabetic status								
Nondiabetic		342 (90.5)		45 (73.8)	0.001∗	Diabetic versus nondiabetic	2.69 (1.18–6.13)	0.018∗∗
Non-insulin dependent		26 (6.9)		11 (18.0)				
Insulin dependent		10 (2.6)		5 (8.2)		Insulin dependent versus non-insulin dependent	4.31 (1.09–16.98)	0.037∗∗
Body mass index (kg/m^2^)								
<25		141 (37.3)		6 (10)	<0.001∗	<25 versus ≥25	2.97 (1.59–5.57)	0.001∗∗
25–29.9		153 (40.5)		27 (45.0)				
≥30		81 (21.4)		27 (45.0)		25–29.9 versus ≥30		0.144
Not recorded		3 (0.8)		1 (1.6)				

∗Significant at the level of 0.25 for univariate analysis and included in multivariate analysis.

∗∗Significant at the level of 0.05 for multivariate analysis.

**Table 2:** Analysis of factors associated with major complications following liver resection in 504 patients.

*N* = 504	No complication (*n* = 410)	Major complication (*n* = 94)	Univariate	Multivariate	
			*P* value	Odds ratio (95% CI)	*P* value
Median age (range)	64 (21–90)	67 (32–88)	0.015∗	1.03 (0.99–1.07)	0.004∗∗
Gender (%)					
Male	211 (51.5)	67 (71.3)	0.001∗	2.36 (1.34–4.17)	0.028∗∗
Female	199 (48.5)	27 (28.7)			
Pathology (%)					
Benign	34 (8.3)	6 (6.4)			
Primary	83 (20.2)	23 (24.5)	0.622		
Secondary	293 (71.5)	65 (69.1)	0.308		
Liver-directed chemotherapy (%)	130 (31.7)	33 (35.1)	0.608		
Preexisting chronic liver disease (%)	10 (2.4)	1 (1.1)	0.666		
Preoperative jaundice (≥50 micromoles/L) (%)	6 (1.5)	3 (3.2)	0.266		
Hypoalbuminaemia (<35 g/L) (%)	9 (2.2)	8 (8.5)	0.004∗	2.97 (1.01–8.74)	0.047∗∗
Raised preoperative alkaline phosphatase (%)	95 (23.2)	24 (25.5)	0.721		
Raised preoperative transaminase (%)	74 (18.0)	21 (22.3)	0.320		
Preoperative glomerular filtration rate (%)					
≤90 mL/min	274 (66.8)	63 (67.0)	0.892		
Median preoperative haemoglobin (g/dL) (range)	13 (9–17)	13 (9–16)	0.025∗		0.439
Median preoperative white cell count (/L) (range)	7 (3–17)	7 (3–25)	0.422		
Diabetic status (%)					
Nondiabetic	370 (90.2)	78 (83.0)	0.014∗		0.912
Non-insulin dependent (versus nondiabetic)	32 (7.8)	9 (9.6)			
Insulin dependent diabetes (versus non-insulin dependent and nondiabetics)	8 (2.0)	7 (7.4)		3.86 (1.17–12.75)	0.028∗∗
Body mass index (kg/m^2^) (%)					
<25	139 (33.9)	28 (29.8)	0.697		
25–30	167 (40.7)	42 (44.7)			
>30	99 (24.1)	24 (25.5)			
American Association of Anesthesiologists (ASA) grade (%)					
1 versus 2			0.198∗		
1	46 (11.2)	6 (6.4)			0.611
2	266 (64.9)	58 (61.7)			
2 versus 3 and 4					
3	95 (23.2)	30 (31.9)			0.783
4	2 (0.5)	0			
Median P-POSSUM physiologic score (range)	16 (12–32)	18 (12–30)	0.003∗		0.764
Operative approach (%)					
Laparoscopic	46 (11.2)	4 (4.3)	0.065∗		0.812
Open	364 (88.8)	90 (95.7)			
Radiofrequency ablation included (%)	18 (4.4)	5 (5.3)	0.698		
Wedge resection included (%)	181 (44.1)	22 (23.4)	<0.001∗		0.353
Bile-duct reconstruction (%)	34 (8.3)	12 (12.8)	0.246∗		0.585
Synchronous bowel procedure (%)	13 (3.2)	10 (10.6)	0.003∗	5.99 (2.25–15.96)	<0.001∗∗
Median number of segments resected (range)	3 (1–6)	4 (1–6)	<0.001	1.51 (1.26–1.80)	<0.001∗∗
Repeat operation (%)	31 (7.6)	6 (6.4)	0.861		
Intraoperative blood loss (%)					
<500 mL	218 (53.2)	29 (30.9)	<0.001		0.463
≥500 mL	188 (45.9)	65 (69.1)			
Blood transfusion required (%)	65 (15.9)	41 (43.6)	<0.001	2.48 (1.44–4.30)	0.001∗∗
Steatosis (%)					
<33%	308 (75.1)	70 (74.5)	1.000		
≥33%	50 (12.2)	11 (11.7)			

∗Significant at the level of 0.25 for univariate analysis and included in multivariate analysis.

∗∗Significant at the level of 0.05 for multivariate analysis.

**Table 3:** Postoperative complications, 90-day mortality, and diabetic status in 504 patients undergoing liver resection (patients may have had more than one complication).

*N* = 504	All patients (*n* = 504)	Nondiabetic (*n* = 448)	Non-insulin dependent diabetes (*n* = 41)	Insulin dependent diabetes (*n* = 15)	*P* value Nondiabetic versus non-insulin dependent diabetic	*P* value Nondiabetic versus insulin dependent diabetic
	Count (%)	Count (%)	Count (%)	Count (%)		
Any major complication	94 (18.7)	78 (17.4)	9 (21.9)	7 (46.6)	0.521	0.010
90-day mortality (Grade V)						
Liver failure	9 (1.8)	5 (1.1)	3	1	0.023∗	0.180
Sepsis	4 (0.8)	3 (0.7)	1	0	0.296	1.000
Malignancy	4 (0.8)	3 (0.7)	0	1	1.000	0.124
Pulmonary embolus	1 (0.2)	1 (0.2)	0	0	1.000	1.000
Anastomotic leak	1 (0.2)	1 (0.2)	0	0	1.000	1.000
Peptic ulcer	1 (0.2)	1 (0.2)	0	0	1.000	1.000
Strangulated hernia	1 (0.2)	1 (0.2)	0	0	1.000	1.000
Peritonitis	1 (0.2)	1 (0.2)	0	0	1.000	1.000
Heart failure	1 (0.2)	1 (0.2)	0	0	1.000	1.000
Total	**23 (4.6)**	**17 (3.8)**	**4**	**2**	**0.089**	**0.122**
Grade IV complications						
Posthepatectomy liver failure (PHLF)	34 (6.7)	30 (6.7)	4	0	0.515	0.613
Renal dysfunction	31 (6.2)	24 (5.4)	4	3	0.280	0.050
Respiratory failure requiring intensive care	2 (0.4)	2 (0.4)	0	0	1.000	1.000
Total	**67 (13.3)**	**56 (12.5)**	**8**	**3**	**0.224**	**0.421**
Grade III complications						
Bile leak						
Drain	12 (2.4)	11 (2.5)	0	1	0.611	0.330
ERCP	11 (2.2)	10 (2.2)	1	0	1.000	1.000
Relaparotomy/relaparoscopy						
Washout	3 (0.6)	1 (0.2)	1	1	0.161	0.064
Adhesiolysis	2 (0.4)	2 (0.4)	0	0	1.000	1.000
Defunction for anastomotic leak	1 (0.2)	0	1	0	0.084	1.000
Small bowel leak	1 (0.2)	1 (0.2)	0	0	1.000	1.000
Drainage						
Liver abscess	1 (0.2)	1 (0.2)	0	0	1.000	1.000
Pleural effusion	1 (0.2)	0	0	1	1.000	0.032
Pneumothorax	1 (0.2)	1 (0.2)	0	0	1.000	1.000
Subphrenic collection	2 (0.4)	2 (0.4)	0	0	1.000	1.000
Total	**35 (6.9)**	**29 (6.5)**	**3**	**3**	**0.743**	**0.077**
